# Spin Magnetic Effect Activate Dual Site Intramolecular O─O Bridging for Nickel‐Iron Hydroxide Enhanced Oxygen Evolution Catalysis

**DOI:** 10.1002/advs.202415525

**Published:** 2025-01-21

**Authors:** Haohai Dong, Lanke Luo, Sitong Zhou, Lin Chen, Xinyu Wu, Yitao Yang, Zhensheng Liao, Liao Fu, Ming Chen, Yuxin Zhu, Peiyuan Su, Haomin Jiang, Zemin Sun, Liu Lin, Qingsong Hua

**Affiliations:** ^1^ Institution Faculty of Arts and Sciences & Center for Advanced Materials Research Beijing Normal University Zhuhai 519087 China

**Keywords:** intramolecular O−O coupling mechanism, NiFe(OH)_2_, oxygen evolution reaction, spin magnetic effect

## Abstract

The oxygen evolution reaction (OER) involves the recombination of diamagnetic hydroxyl (OH) or water (H_2_O) into the paramagnetic triplet state of oxygen (O_2_). The spin conservation of oxygen intermediates plays a crucial role in OER, however, research on spin dynamics during the catalytic process remains in its early stages. Herein, *β*‐Ni(OH)_2_ and Fe‐doped *β*‐Ni(OH)_2_ (Ni_5_Fe_1_(OH)_2_) are utilized as model catalysts to understand the mechanism of spin magnetic effects at iron (III) sites during OER. Combined with magnetic characterization, it is founded that the introduction of Fe transforms the antiferromagnetic Ni(OH)_2_ into a ferromagnetic material. Testing the magnetic response of the catalyst under an external magnetic field, the OER activity of Ni_5_Fe_1_(OH)_2_ is significantly enhanced in comparison to Ni(OH)_2_. This improvement is likely due to the introduction of iron sites, which promote spin magnetic effects and enhance reaction kinetics, thereby increasing catalytic efficiency. Combining experimental and theoretical characterization, it is discovered that the iron sites accelerate the formation of heterogeneous dual‐site O─O bridging, represented as ─Ni─O─O─Fe─, thereby effectively enhancing the kinetics of the OER reaction. This study provides a magnetic perspective on the structure‐function relationship of magnetic iron‐based catalysts and has significant implications for the design of new catalysts.

## Graphical abstract

The magnetic response test of Ni_5_Fe_1_(OH)_2_ indicates that its OER activity is superior to that of Ni(OH)_2_. Under magnetic field conditions, the iron site accelerates the formation of O─O bridge, denoted as ─Ni─O─O─Fe─, which effectively enhances the kinetics of the OER. This study provides a magnetic perspective on the structure‐function relationship of iron‐based catalysts, offering valuable insights for the design of new catalysts.

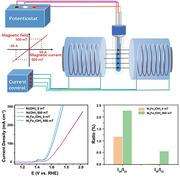

## 1 Introduction

The kinetics of the oxygen evolution reaction (OER) are relatively slow due to the complex four‐electron process involving the breaking and reformation of O─H bonds.^[^ ^1^ ^]^ This reaction is critically important for achieving carbon neutrality, as it is a key half‐reaction in water splitting and rechargeable metal‐air batteries.^[^ ^2^ ^]^ During the OER process, the initial reactants, H_2_O/OH^−^, are in a singlet state with all electrons paired, while the final product, triplet oxygen, has two parallel unpaired electrons. Therefore, the induction of spin rearrangement requires a change in energy barrier.^[^ ^3^ ^]^ However, the spin interactions between the catalyst and oxygen intermediates are crucial for spin conservation, yet this aspect is often overlooked.^[^ ^4^ ^]^ The application of spin effects in oxygen catalysis remains a relatively unexplored area.

Recently, significant efforts have been made to develop high‐performance OER catalysts, among which nickel‐iron‐based hydroxide catalysts (NiFe(OH)_2_) are widely regarded as benchmark catalysts for OER in alkaline media.^[^ ^5^ ^]^ Although the high catalytic activity of NiFe(OH)_2_ has been well established, the origin of this activity and the enhancement mechanism introduced by iron remains controversial.^[^ ^6^ ^]^ The magnetic characteristics of iron sites and their spin magnetic response mechanisms during the OER catalytic process have not been systematically studied, limiting the understanding of nickel‐iron‐based hydroxide catalysts.^[^ ^7^ ^]^ During the OER reaction, the magnetic field can act as a “magnifying glass,” amplifying the spin interactions during the reaction process and providing a novel perspective for understanding the internal spin effects.

Inspired by these reasons, we synthesized Ni(OH)_2_ and Ni_5_Fe_1_(OH)_2_ nanosheets as model structures to understand the mechanism of spin magnetic effects at Fe sites. Compared to Ni(OH)_2_, the presence of iron sites can effectively activate the spin magnetic effect. By combining magnetism, spectra, and theoretical calculations, we discovered for the first time that the spin magnetic effect of the iron sites can accelerate the lattice oxygen‐mediated mechanism (LOM), facilitating the formation of the ─Ni─O─O─Fe─ oxygen bridge, thereby improving the OER reaction kinetics. This study proposes that spin magnetic effects could promote the formation of heterogeneous dual‐site intramolecular O─O bridging, further understanding the lattice oxygen‐mediated mechanism LOM mechanism for heterogeneous atomic catalysts. This work also provides a magnetic perspective on the structure‐function relationship of magnetic iron‐based catalysts and is beneficial for the design of new catalysts.

## 2 Results and Discussion

### 2.1 Structural Characterization of Ni(OH)_2_ and Ni_5_Fe_1_(OH)_2_

**Figure 1:**
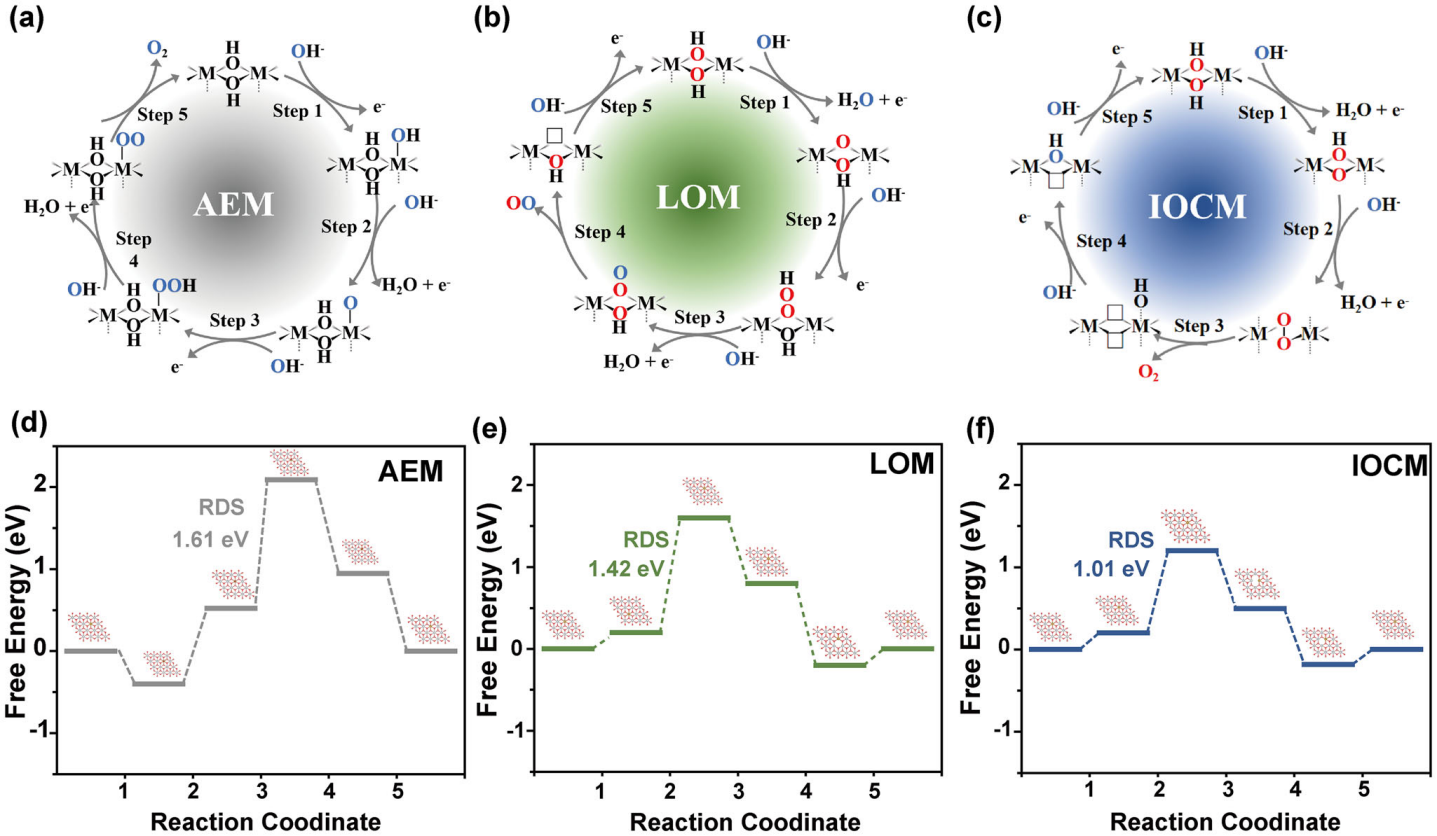
OER mechanisms of (a) AEM, (b) LOM, and (c) IOCM; Free energy diagrams of intermediates in OER on Ni_5_Fe_1_(OH)_2_ model for the (d) AEM, (e) LOM and (f) IOCM, respectively.

In the oxygen evolution reaction (OER), researchers have proposed three main mechanisms, as illustrated in **Figure** **1**a–c: the adsorbate evolution mechanism (AEM), the lattice oxygen‐mediated mechanism (LOM), and the intramolecular ^*^O−^*^O coupling mechanism (IOCM).^[^ ^8^ ^]^ By constructing a structural model of Ni_5_Fe_1_(OH)_2_ (Figure S1, Supporting Information) and considering the reaction pathways of AEM (Figure 1d), LOM (Figure 1e), and IOCM (Figure 1f), the overpotentials for these three mechanisms were calculated using density functional theory (DFT). To better understand the mechanism of OER at the iron site, the rate‐determining step (RDS) in the AEM process has been transformed into the oxidation of OH to OOH at the third step, with an energy barrier of 1.61 eV. In contrast to the AEM mechanism, the LOM emphasizes the creation of oxygen vacancies on the catalyst surface as a means of surface oxygen evolution. The OH adsorbed by Fe(OH)_6_ deprotonates to form O, which then accepts OH^−^ through nucleophilic attack to form OOH. Subsequently, OOH deprotonates to generate OO, releasing O_2_ to create an oxygen vacancy, which is filled by OH^−^. Similarly, the formation of ^*^OOH is the RDS with an energy barrier of 1.42 eV. For the IOCM process, the reaction pathway involves the successive adsorption and oxidation of OH^−^ on by Fe(OH)_6_ and Ni(OH)_6_ deprotonates to form O, and then leading to the formation of a heterogeneous Ni─O─O─Fe structure. The intermediate material is then gradually oxidized, ultimately producing oxygen. Compared to the energy barrier of 1.42 eV that needs to be overcome in the unit point catalytic pathway, this pathway's energy barrier is reduced to 1.01 eV. This indicates that the synergistic catalytic pathway of Fe and Ni bimetals significantly promotes electron transfer, thereby accelerating the kinetics of the OER reaction. Based on this, the key to the RDS in the three reaction processes is the formation of the ─O─O─ bond, and dual site intramolecular O─O bridging can effectively lower the reaction energy barrier.

**Figure 2:**
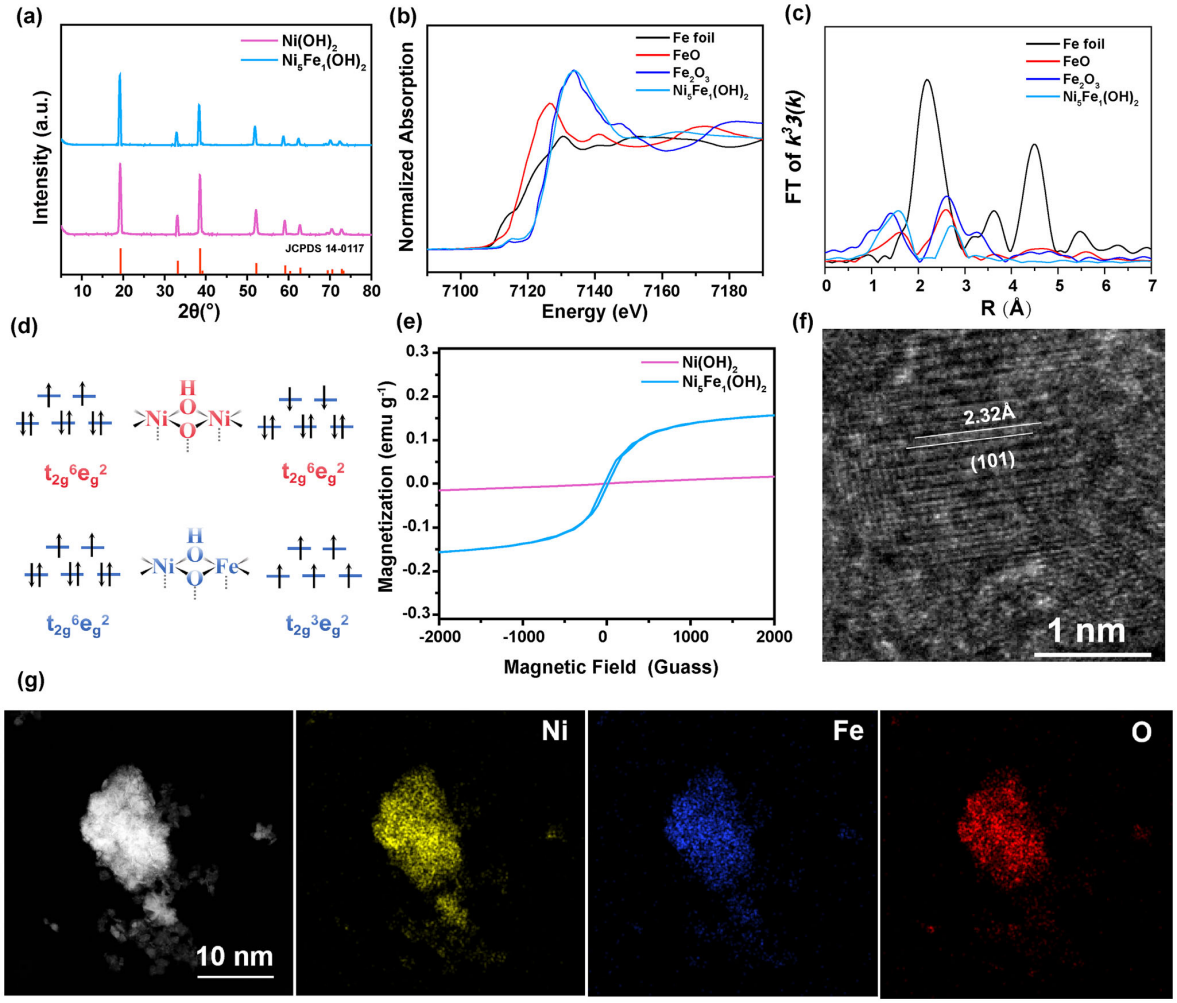
a) XRD of Ni(OH)_2_ and Ni_5_Fe_1_(OH)_2_; b) Fe K‐edge XAFS spectra of Ni_5_Fe_1_(OH)_2_; c) R‐space plots of Ni_5_Fe_1_(OH)_2_; d) The spin state of Ni^2+^ and Fe^3+^ in Ni(OH)_2_ and Ni_5_Fe_1_(OH)_2_; e) VSM of Ni(OH)_2_ and Ni_5_Fe_1_(OH)_2_; f) The HRTEM of Ni_5_Fe_1_(OH)_2_; g) The energy‐dispersive spectrometry (EDS) mapping of Ni, Fe and O of the Ni_5_Fe_1_(OH)_2_.

Based on the theoretical calculation results, the model catalyst Ni_5_Fe_1_(OH)_2_ and its blank control Ni(OH)_2_ were prepared via a co‐precipitation method, with and without Fe^3+^ metal salts, respectively. As shown in **Figure** **2**a, the *β*‐Ni(OH)_2_ sample exhibited diffraction peaks at 2θ = 19.3°, 33.1°, 38.5°, 59.1°, and 62.7°, corresponding to the (111), (200), (220), (311), and (222) planes of the hexagonal *β*‐Ni(OH)_2_ phase material (JCPDS 14‐0117). The diffraction peaks of the NiFe(OH)₂ sample were consistent with the *β*‐Ni(OH)_2_ lattice, with no additional impurity peaks observed. For Ni(OH)_2_ and Ni_5_Fe_1_(OH)_2_, the ionic radii of Fe^3+^ ions (with an ionic radius of ≈0.645 Å) and Ni^2+^ ions (with an ionic radius of ≈0.69 Å) are different. When Fe^3+^ partially substitutes for Ni^2+^ to form Ni_5_Fe_1_(OH)_2_, due to the smaller ionic radius of Fe^3+^, the crystal lattice will contract. From the perspective of interplanar spacing, according to Bragg's Law, the interplanar spacing d is inversely proportional to the diffraction angle θ. When the crystal lattice contracts, the interplanar spacing d decreases. As a result, the value of sinθ increases, thereby causing the diffraction angle to shift toward higher angles. The inductively coupled plasma atomic emission spectroscopy (ICP‐AES) analysis indicated a molar ratio of Fe to Ni of 1:5, as shown in Table S1 (Supporting Information), which is consistent with the molar ratios used in the reaction, confirming the successful synthesis of the Ni_5_Fe_1_(OH)_2_ model catalysts. Further analysis of the chemical properties and electronic structure of the Ni(OH)_2_ and Ni_5_Fe_1_(OH)_2_ were conducted using X‐ray photoelectron spectroscopy (XPS). As shown in Figure S2 (Supporting Information), the Ni 2p spectrum of Ni(OH)_2_ exhibited two peaks at 873.2 eV and 855.5 eV, corresponding to the binding energies of Ni 2p_1/2_ and Ni 2p_3/2_, respectively. Upon the introduction of Fe^3+^, the two peaks for Ni 2p_3/2_ and Ni 2p_1/2_ displayed slight negative shifts toward higher binding energies, which is consistent with the literature.^[^ ^2h^ ^]^ The high‐resolution Fe 2p spectrum of Ni_5_Fe_1_(OH)_2_ revealed binding energies of Fe^3+^ 2p_3/2_ and Fe^3+^ 2p_1/2_ at 707.13 and 713.17 eV, respectively, as shown in Figure S3 (Supporting Information), further confirming the successful incorporation of Fe^3+^ into the Ni(OH)_2_.^[^ ^9^ ^]^ To elucidate the atomic structure and local coordination of Ni_5_Fe_1_(OH)_2_, we employed X‐ray absorption spectroscopy (XAS). As shown in Figure 2b, the valence state of Fe in Ni_5_Fe_1_(OH)_2_ is closed to +3, consistent with the XPS result. In the Fe K‐edge FT‐EXAFS presented in Figure 2c, the absence of an Fe─Fe bond at ≈2.20 Å confirms the atomic dispersion of Fe within the Ni(OH)_2_ layers in both samples, ruling out the formation of Fe clusters or nanoparticles. Furthermore, the presence of the Fe─O bond and Fe─O─Ni bond at 1.60 and 2.48 Å, respectively.^[^ ^10^ ^]^ The Fe─O─Ni bond demonstrates the interaction between Fe and Ni atoms in both samples, representing the coordination in the second shell.^[^ ^11^ ^]^

The magnetic properties were further measured using a vibrating sample magnetometer (VSM), as shown in Figure 2d,e. For the Ni(OH)₂, Ni^2+^ exists in the t_2g_^6^e_g_^2^ configuration, where the spin state of Ni^2+^ on both sides of the oxygen exhibits a reverse symmetric structure, resulting in an overall antiferromagnetic behavior. The introduction of Fe^3+^ (t_2g_^3^e_g_^2^) alters the symmetry of the spin electrons within the layered structure, transforming its magnetic behavior from antiferromagnetism to ferromagnetism. This suggests that the electrocatalytic OER activity of Ni_5_Fe_1_(OH)_2_ might possess spin magnetic responsive characteristics compared to Ni(OH)_2_. Morphological characterizations of Ni(OH)_2_ and Ni_5_Fe_1_(OH)_2_ were conducted using scanning electron microscopy (SEM) and transmission electron microscopy (TEM), with results displayed in Figures S4 and S5 (Supporting Information) showing that both Ni(OH)_2_ and Ni_5_Fe_1_(OH)_2_ exhibit uniform ultrathin nanosheet structures. To further confirm the nanosheet structure of Ni(OH)₂ and Ni_5_Fe_1_(OH)_2_, high‐resolution transmission electron microscopy (HRTEM) was employed. Figure 2f and Figure S6 (Supporting Information) indicate that the lattice spacing decreases due to differences in ionic radius before and after Fe^3+^ doping, consistent with XRD results. Additionally, elemental mapping, as shown in Figure 2g, reveals a uniform distribution of Ni, Fe, and O elements on the nanosheet surfaces. These results collectively demonstrate the successful incorporation of Fe into the lattice, thereby constructing an ideal model structure.^[^ ^2h^ ^]^

### 2.2 Spin Magnetic Effect of Ni(OH)_2_ and Ni_5_Fe_1_(OH)_2_

The OER activity of the obtained catalyst was evaluated in O_2_‐saturated 1.0 M KOH solution. The OER polarization curves of the prepared samples are shown in Figure S7 (Supporting Information). The Ni_5_Fe_1_(OH)_2_ electrode exhibited an overpotential of only 299 mV at 20 mA cm^−2^, significantly lower than the 349 mV observed for Ni(OH)_2_. Tafel plots were utilized to assess the catalytic reaction kinetics (Figure S8, Supporting Information), revealing a Tafel slope of 139.6 mV dec^−1^ for Ni_5_Fe_1_(OH)_2_, compared to 262.9 mV dec^−1^ for Ni(OH)_2_, indicating that the introduction of iron enhances the catalytic kinetics. Additionally, electrochemical impedance spectroscopy (EIS) Nyquist plots were recorded to investigate charge transfer behavior, as shown in Figure S9 (Supporting Information). The impedance of Ni_5_Fe_1_(OH)_2_ was significantly lower than that of Ni(OH)_2_, suggesting that the developed electrode exhibits more favorable charge transfer dynamics during the OER process. Furthermore, the electrochemical active surface area (ECSA) of the catalyst in Figure S10 (Supporting Information) was calculated based on the double‐layer capacitance (C_dl_) values (Figure S11, Supporting Information). The results indicate a steady increase in ECSA with the introduction of iron. Combining structural and performance testing results, it was found that the introduction of iron can transform the catalyst from antiferromagnetic to ferromagnetic. Whether the unique magnetic properties of the iron sites contribute to the enhanced OER activity remains to be further explored. Besides, the XPS of Ni(OH)_2_ and Ni_5_Fe_1_(OH)_2_ after electrochemical testing as shown in Figure S12 (Supporting Information) can verify that the two peaks of Ni 2p_3/2_ and Ni 2p_1/2_ shift slightly toward the higher binding energy direction, which implies the generation of Ni^3+^ species and is consistent with the literature.^[^ ^2h^ ^]^

**Figure 3:**
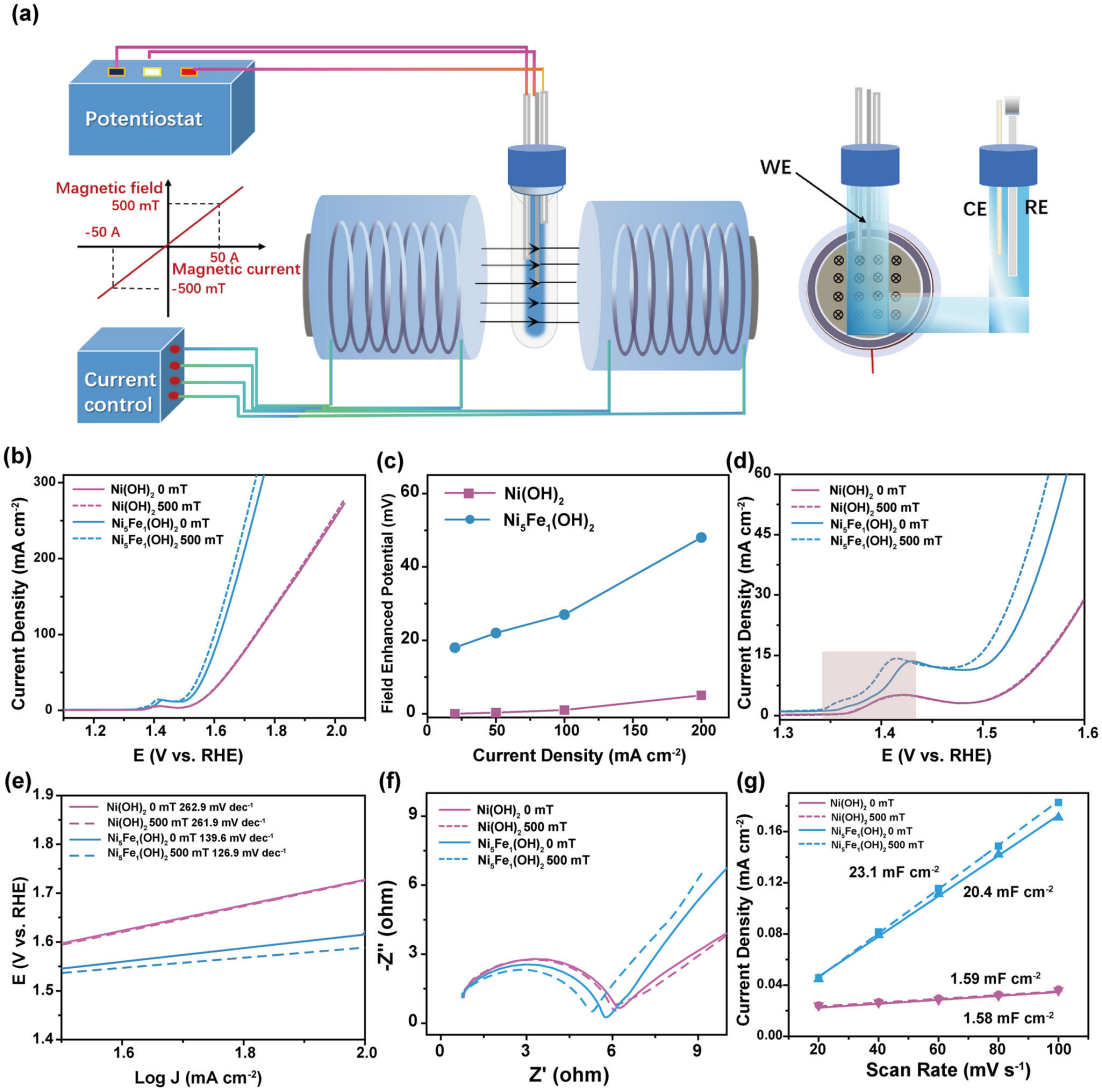
a) Magnetic field‐electrocatalysis combined device; b) The LSV of Ni(OH)_2_ and Ni_5_Fe_1_(OH)_2_ under 0 and 500 mT; c) The reduced overpotential with magnetic field effect; (c) The LSV of Ni(OH)_2_ and Ni_5_Fe_1_(OH)_2_ under 0 and 500 mT; d)The redox peak of Ni(OH)_2_ and Ni_5_Fe_1_(OH)_2_ under 0 and 500 mT; e) Tafel slope, f) EIS and g) ECSA of Ni(OH)_2_ and Ni_5_Fe_1_(OH)_2_ under 0 and 500 mT.

To understand the spin magnetic effects of Fe^3+^ in Ni_5_Fe_1_(OH)_2_, the electrocatalytic performance of Ni(OH)_2_ and Ni_5_Fe_1_(OH)_2_ was further tested under a 500 mT magnetic field using a vibrating sample magnetometer (VSM) in conjunction with electrochemical measurements (**Figure** **3**a). As shown in Figure 3b, the LSV curves indicate changes in both Ni(OH)_2_ and Ni_5_Fe_1_(OH)_2_ upon the introduction of the magnetic field; however, the performance variations differ between the two. Analyzing the current densities under different potential conditions as shown in Figure 3c and Figures S13 and S14 (Supporting Information) reveals that Ni(OH)_2_ exhibited almost no change at low current densities, with only a slight increase at high current densities, primarily due to the magnetic field accelerating the diffusion dynamics of oxygen molecules, enhancing mass transfer kinetics. The introduction of Fe^3+^ facilitated the transition of Ni(OH)_2_ from antiferromagnetic to ferromagnetic. At high current densities, the OER activity of Ni_5_Fe_1_(OH)_2_ was also improved, attributed to enhanced mass transfer and diffusion effects from the magnetic field. Notably, compared to Ni(OH)₂, the magnetic response of Ni_5_Fe_1_(OH)_2_ enhanced catalytic activity, resulting in an increased current density at low potentials. Combining the details from Figure 3d, it can be observed that the magnetic field effectively lowered the onset potential of Ni_5_Fe_1_(OH)_2_. These results indicate that the magnetic field could significantly influence the iron sites, optimizing the reaction process and enhancing reaction kinetics. Further analysis of the system's kinetics was conducted using the Tafel slope and EIS measurements. As shown in Figure 3e, the Tafel slope of the antiferromagnetic Ni(OH)_2_ remained almost unchanged under the influence of the magnetic field, while the Tafel slope of the ferromagnetic Ni_5_Fe_1_(OH)_2_ decreased from 139.6 to 136.9 mV dec^−1^, which suggests that the magnetic field could promote the kinetics of the OER reaction. To assess the field‐assisted electron transfer capability, the corresponding EIS curves were measured in Figure 3f. It demonstrates that the impedance of Ni(OH)_2_ did not exhibit significant changes under a 500 mT magnetic field. In contrast, the impedance of Ni_5_Fe_1_(OH)_2_ was significantly reduced, further indicating that the magnetic field enhances the electron transport efficiency and interfacial reaction kinetics of Ni_5_Fe_1_(OH)_2_. Additionally, by comparing the ECSA of Ni(OH)₂ and Ni_5_Fe_1_(OH)_2_ under 0 and 500 mT, the effective active sites of the catalyst increased from 20.4 to 23.1 mF cm^−2^ (Figure 3g and Figure S15, Supporting Information), while changes in Ni(OH)₂ were negligible. This further indicates that the magnetic field might enhance and activate new active sites, thereby influencing the OER process and improving reaction kinetics.

### 2.3 Spin Magnetic Effect of Ni(OH)_2_ and Ni_5_Fe_1_(OH)_2_

**Figure 4:**
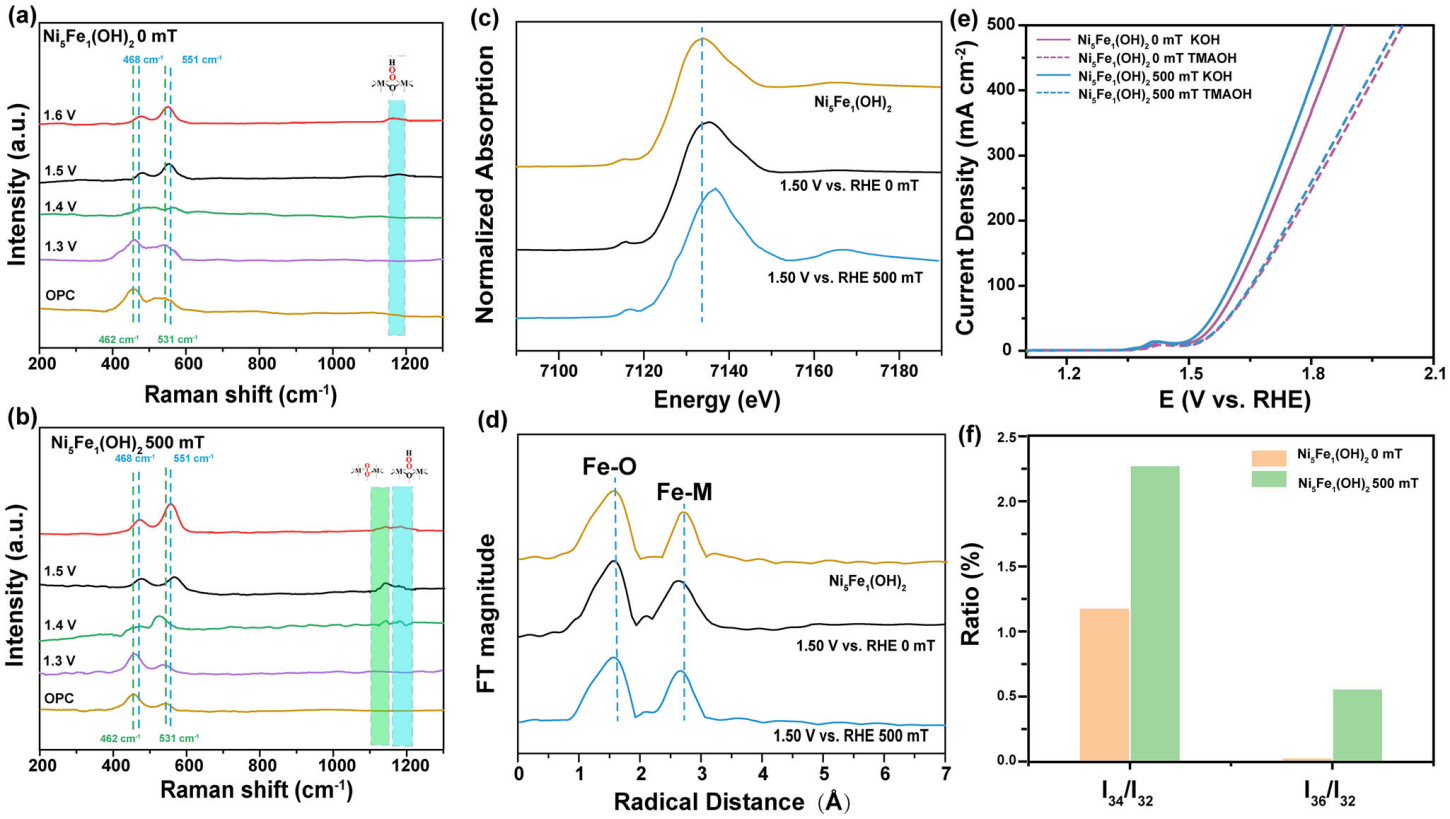
In situ Raman spectroscopy of a) Ni_5_Fe_1_(OH)_2_ under 0 mT and b) Ni_5_Fe_1_(OH)_2_ under 500 mT.c) XANES spectra and d) Fe‐K edge EXAFS spectra of the Fe‐K edge on Ni_5_Fe_1_(OH)_2,_Ni_5_Fe_1_(OH)_2_ at 1.5 V vs. RHE under 0 mT and Ni_5_Fe_1_(OH)_2_ at 1.5 V vs. RHE under 500 mT. e) LSV curves of Ni_5_Fe_1_(OH)_2_ under 0 and 500 mT in 1 m KOH and 1 m TMAOH; f) Accumulation of gas chromatographic signals of ^18^O isotope labeled oxygen products for 15 min.

To better understand the intrinsic mechanism of the magnetic field's influence on the oxygen evolution reaction (OER) during the reaction process, we conducted a series of characteristic experiments. Operando electrochemical Raman spectroscopy effectively monitors the intermediates in surface OER processes under both magnetic and non‐magnetic conditions. Initially, we performed in situ electrochemical surface‐enhanced Raman scattering (SERS) measurements to track the OER process on the surface of Ni_5_Fe_1_(OH)_2_. As shown in **Figure** **4**a, Raman spectra were collected at open‐circuit potential (OCP), with a peak at 462/531 cm^−1^ attributed to the stretching mode of *β*‐Ni(OH)_2_. Upon further increasing the potential to 1.4 V, a new broad‐shouldered 557 cm^−1^, corresponding to the stretching mode of the Ni─O bond in NiOOH, indicating that the phase transition at the Ni site begins. It is consistent with the oxidation peak potential of LSV. Until the potential reaches 1.5 V, the *γ*‐NiOOH peaks appear as shoulders at 468 and 551 cm^−1^. Furthermore, a broadened peak appeared at 1150 cm^−^¹, likely due to the formation of the Ni─O─O─Fe bond, where O─O stretching vibrations of top‐mode adsorptions, demonstrating the LOM process.^[^ ^8^, ^12^ ^]^ When a magnetic field was applied (Figure 4b), a new peak at 1151.1 cm^−^¹ appeared at 1.3V, suggesting that the magnetic field acts on the Fe sites to promote the formation of Ni─O─O─Fe, the bands 1139.2 and 1153.9 cm^−1^ could be reasonably assigned to O─O stretching vibrations for bridge and top‐mode adsorption, it might follow ICOM mechanism.^53^ By comparing Figure 4a,b, it is further proved that the 500 mT magnetic field effectively promotes the formation of Ni─O─O─Fe Bridging in Fe‐Ni(OH)_2_. X‐ray absorption spectroscopy (XAS) was employed to study the local chemical structure around the Fe centers before and after activation. When the potential is increased to 1.50V as shown in Figure 4c, both the leading edge peak and the main absorption edge of the Fe K‐edge XANES spectra shift significantly, indicating that the Fe site is oxidized, and the oxidation reconstruction effect would be enhanced under the magnetic field, which is consistent with the results of Raman spectroscopy. By fitting the X‐ray absorption near‐edge structure (XANES) spectrum at the Fe K‐edge in Figure 4d, we revealed two main peaks of Fe appear at 2.00 Å and 3.04 Å, corresponding to Fe─O and Fe─Fe bond. Both the Fe K‐edge spectra at 1.5 V vs. RHE exhibit significant bond contraction, which should be attributed to the high‐valence Fe─O─O─Ni species and might contribute to the high OER performance. It is worth noting that the magnetic field catalyzes, and the shrinkage is more severe, further suggesting the difference between the two lattice oxygen mechanisms. Similarly, the local chemical structure around the Ni site was also characterized as shown in Figure S16 (Supporting Information). When the potential increased to 1.50 V, the leading edge peak and main absorption edge of the Ni K ‐ edge XANES spectra shifted significantly regardless of the presence of a magnetic field (Figure S16a, Supporting Information), indicating that Ni was oxidized. By fitting the XANES spectra of the Ni K ‐ edge in Figure S16b (Supporting Information), we found that its Ni─O and Ni─Fe/Ni bonds both showed significant bond contraction. It is worth noting that the magnetic field has a relatively small effect on the Ni site compared to the Fe site, further suggesting that the magnetic field mainly affects the Fe site and thus influences the catalytic activity.

As shown in Figure S17 (Supporting Information), Ni_5_Fe_1_(OH)_2_ exhibited more pronounced pH‐dependent kinetics in the presence of a magnetic field, indicating that the magnetic field facilitates the formation of Fe─O─O─Ni oxygen bridge bonds, participating in the OER process. Using tetramethylammonium cation (TMA^+^) as a chemical probe, we chemically detected peroxidic species (O_2_^2−^) to seek indirect confirmation of the proposed magnetic field‐enhanced Ni─O─O─Fe pathway. As shown in Figure 4e, the introduction of TMA⁺ led to a significant decrease in OER activity, both with and without a magnetic field, bringing both cases close to the same level. This further suggests that the magnetic field enhances OER kinetics by promoting the peroxy bond of heterogeneous atoms in Ni_5_Fe_1_(OH)_2_. In situ ^18^O isotope labeling combined with differential electrochemical mass spectrometry (DEMS) was used to ascertain the involvement of lattice oxygen in the OER process. Figure 4f displays the detection results of ^16^O^16^O, ^18^O^16^O, and ^18^O^18^O in the OER products of the Ni_5_Fe_1_(OH)_2_ electrode. The presence of ^18^O^16^O and ^18^O^18^O indicates the participation of lattice oxygen in the reaction. Importantly, the signal for ^18^O^18^O suggests that two activated lattice oxygen directly couple to form a single oxygen molecule. To further verify the generation of ^18^O^18^O, the introduction of a magnetic field increased the amount of ^18^O^18^O in the product, indicating that the magnetic field can enhance the direct coupling of two lattice oxygens to form oxygen molecules, thereby reducing overpotential. Through experimental and theoretical methods, this study further elucidates how the introduction of Fe enables the formation of heterogeneous atomic oxygen bridging bonds, accelerating the OER process, while the applied magnetic field facilitates the formation of the Ni─O─O─Fe bond at the iron site, following ICOM process, thereby providing further experimental evidence for our deeper understanding of spin magnetic effects.

## 3 Conclusion

In conclusion, we present new insights into how spin magnetic effects enhance the OER. DFT simulations and spectroscopic analyses confirm that the OER pathway for Ni_5_Fe_1_(OH)_2_ follows a heterogeneous dual‐site O─O bridging mechanism, which effectively reduces reaction overpotential and improves kinetics. By comparing Ni(OH)_2_ and Ni_5_Fe_1_(OH)_2_, we explored the role of spin magnetic effects at the Fe^III^ site. The magnetic field influences the iron site in Ni_5_Fe_1_(OH)_2_, promoting the formation of the Ni─O─O─Fe structure at very low potentials. This comprehensive understanding of the OER mechanism is crucial for guiding the design of multiphase dual‐site electrocatalysts and advancing the development of effective electrocatalytic materials.

## Conflict of Interest

The authors declare no conflict of interest.

## Supporting information



Supporting Information

## Data Availability

Research data are not shared.
