# Correction to “Spin Magnetic Effect Activate Dual Site Intramolecular O‐O Bridging for Nickel‐Iron Hydroxide Enhanced Oxygen Evolution Catalysis”

**DOI:** 10.1002/advs.202512423

**Published:** 2025-08-13

Dong HH, Luo LK, Zhou ST, et al., Spin Magnetic Effect Activate Dual Site Intramolecular O‐O Bridging for Nickel‐Iron Hydroxide Enhanced Oxygen Evolution Catalysis. *Adv Sci (Weinh)*. **2025**;*12* (10): e2415525.

https://doi.org/10.1002/advs.202415525

Description of the errors:

The model diagram of the magnetic field‐electrocatalysis device in Figure 3A of the paper was borrowed and adapted from Figure 2 of Nat. Commun. 2024, 15, 2867. Since the original work was not cited for copyright, we hereby make a correction by adding the citation to avoid copyright issues.

We have added Reference [12]:

[12] P. Vensaus, Y. Liang, J. Ansermet, G. Soler‐Illia, M. Lingenfelder, *Nat. Commun*. **2024**, *15*, 2867.

Consequently, the original Reference [12] is renumbered as Reference [13]:

[13] a) D. K. Cho, H. W. Lim, A. Haryanto, B. Yan, C. W. Lee, J. Y. Kim, *ACS Nano* **2024**, *18*, 20459−20467; b) C. Hu, Y. Hu, C. Fan, L. Yang, Y. Zhang, H. Li, W. Xie, *Angew. Chem. Int. Ed*. **2021**, *60*, 19774−19778.


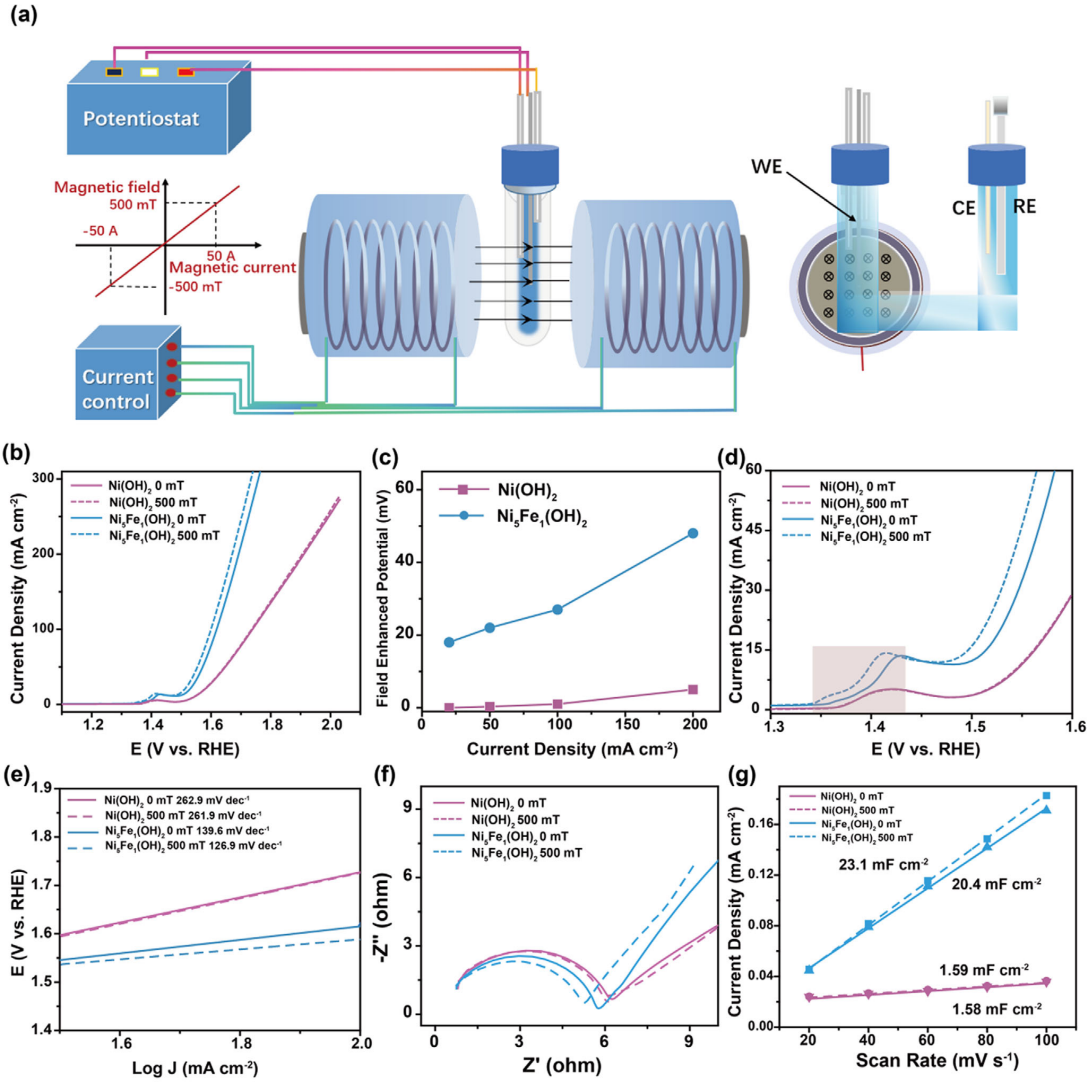


**Figure 3**. (a) Magnetic field‐electrocatalysis combined device; Reproduced with permission [12]. Copyright 2024, Springer Nature. (b) The LSV of Ni(OH)_2_ and Ni_5_Fe_1_(OH)_2_ under 0 mT and 500 mT; (c) The reduced overpotential with magnetic field effect; (c) The LSV of Ni(OH)_2_ and Ni_5_Fe_1_(OH)_2_ under 0 mT and 500 mT; (d)The redox peak of Ni(OH)_2_ and Ni_5_Fe_1_(OH)_2_ under 0 mT and 500 mT; (e) Tafel slope, (f) EIS and (g) ECSA of Ni(OH)_2_ and Ni_5_Fe_1_(OH)_2_ under 0 mT and 500 mT.

