# Supplementary material for: Spin Magnetic Effect Activate Dual Site Intramolecular O─O Bridging for Nickel‐Iron Hydroxide Enhanced Oxygen Evolution Catalysis
Source: Adv Sci (Weinh). 2025 Jan 21;12(10):2415525. doi: 10.1002/advs.202415525 (PMC11904962; doi:10.1002/advs.202415525)
Supplement: Supplementary file 1 — Supporting Information [file ADVS-12-2415525-s001.docx]

**Supplementary Information**

Spin Magnetic Effect Activate Dual Site O-O Bridging for Nickel-Iron Hydroxide Enhanced Oxygen Evolution Catalysis

Haohai Dong,^#^ Lanke Luo,^#^ Sitong Zhou,^#^ Lin Chen,* Xinyu Wu, Yitao Yang, Zhensheng Liao, Liao Fu, Ming Chen, Yuxin Zhu, Peiyuan Su, Haomin Jiang, Zemin Sun,* Liu Lin,* and Qingsong Hua*

Faculty of Arts and Sciences & Center for Advanced Materials Research, Beijing Normal University, Zhuhai 519087, China

1. mail addresses: linchen@bnu.edu.cn (L. Chen); linliu@mail.bnu.edu.cn (L. Lin); zmsun@mail.bnu.edu.cn (Z. M. Sun); q.hua@bnu.edu.cn (Q. S. Hua)

^#^ These authors contributed equally to this work.

Experimental method

Synthesis of Ni(OH)₂ and Ni_5_Fe_1_(OH)_2_

The hydrothermal preparation of *β*-Ni(OH)_2_ generally entails dissolving 10 mmol of Ni(NO_3_)_2_·6H_2_O in deionized water to formulate a solution with the requisite concentration. Subsequently, 0.5 g of NaOH is incorporated to adjust the pH value to around 12, thereby facilitating the formation of hydroxide. The resultant mixed solution is transferred into a sealed stainless steel autoclave and subjected to a reaction at a designated temperature of 140 °C for 6 hours in the presence of carbon paper, which aids in the crystallization of β-Ni(OH)₂. Upon completion of the reaction, the system is permitted to cool down to ambient temperature, and the precipitate is gathered and rinsed multiple times with deionized water to eliminate impurities and unreacted precursors. Eventually, the washed product is dried in either a vacuum or a constant-temperature oven to procure the final *β*-Ni(OH)_2_ powder. In the aforementioned reaction sequence, when 2 mmol of Fe(NO)_3_·9H_2_O is added while keeping other conditions unaltered, Ni_5_Fe_1_(OH)_2_ is obtained.

Characterizations

X-ray diffraction (XRD) measurements were performed using a Phillips X'pert ProMPD diffractometer with Cu Kα radiation (λ=1.54056 Å). The generator was set to 40 kV and 40 mA. Field emission scanning electron microscopy (FESEM) images were obtained using a Hitachi scanning electron microscope with an acceleration voltage of 10 kV. High-resolution transmission electron microscopy (HRTEM) measurements were conducted using a FEI Tecnai F20 transmission electron microscope with an acceleration voltage of 200 kV. X-ray photoelectron spectroscopy (XPS) measurements were carried out using an ESCALAB 250Xi spectrometer (Thermo Fisher) with Al Kα radiation. The magnetization measurements were conducted on a Quantum Design Physical Property Measurement System (PPMS). Inductively Coupled Plasma Optical Emission Spectroscopy (ICP-OES) was performed on a PerkinElmer AVIO 500 spectrometer. X-ray absorption spectrum (XAS) measurement of Fe K-edge were recorded in transmission mode and the obtained X-ray absorption near-edge structure (XANES) data was analyzed through the Athena programs. The extended X-ray absorption fine structure (EXAFS) region was k2-weighted and Fourier-transformed in k-ranges of 0-6.0 Å. The operando XAFS was collected based on the Self-made stainless-steel mold.

Electrochemical measurements.

An electrochemical workstation (CHI 760E, Shanghai Chenhua, China) was used to perform electrochemical tests in a standard three-electrode system. The catalysts (0.5×0.5 cm), Hg/HgO electrode, and graphite electrode were used as the working electrode, reference electrode, and counter electrode, respectively. OER tests were performed in 1 M KOH electrolyte. All potentials of the catalyst were converted to the potential of the reversible hydrogen electrode (RHE) according to the Nernst equation: E_RHE_ = E_Hg/HgO_+0.098+0.059×pH and the overpotential at a current density of 10 mA·cm^-2^ was calculated according to the equation: η_10_ = E_RHE_-1.23V. Linear scanning voltammetry (LSV) was performed over a potential range of 0.105 to 1.105 V (vs. Hg/HgO) with a scan rate of 5 mV·s^–1^. Measurements of the double layer capacitance (C_dl_) were carried out by cyclic voltammetry (CV) in a potential window of 0.25-0.35 V (vs. Hg/HgO) at scan rates of 20, 40, 60, 80, and 100 mV·s^–1^. The electrochemically active surface area (ECSA) of the synthesized electrocatalysts was estimated according to the equation: ECSA=C_dl_/C_s_, and C_s_ is the specific capacitance of the corresponding surface smoothed samples under the same conditions. Electrochemical impedance spectroscopy (EIS) was performed at open-circuit voltage and at frequencies ranging from 0.01 Hz to 1000 kHz with a sinusoidal amplitude of 5 mV. DEMS measurements were conducted using a QAS 100 device (Linglu Instruments, Shanghai). and the ^18^O-labeled Ni_5_Fe_1_(OH)_2_ in a standard three-electrode system, CV measurements were performed in KOH solution using H_2_^16^O, with a scan rate of 5 mV·s^–1^. Simultaneously, gas products of varying molecular weights were detected in real-time using mass spectrometry.

Computational details.

DFT calculations were performed using the spin-polarized Vienna Ab initio Simulation Package (VASP) with a periodic plane wave framework. The Perdew-Burke-Ernzerhof (PBE) flavor of the generalized gradient approximation (GGA) was employed. The kinetic energy cutoff for the plane-wave basis set was set to 550 eV, and core electrons were represented by the projector augmented wave (PAW) method. The theoretical model is based on a monolayer of *β*-phase derived Ni(OH)_2_ with a (100) orientation, constructing the Ni_5_Fe_1_(OH)_2_ structure. To prevent interactions between systems, a vacuum layer of at least 15 Å was introduced along the (100) edge and the vertical direction.

The Gibbs free energy of each reaction intermediate is defined as:

G=E_DFT_+E_ZPE_+H−TS

where E_DFT_ is the electronic energy calculated by DFT; E_ZPE_, H, and TS represent the zero-point energy obtained from vibrational frequencies, enthalpy, and entropy, respectively. It is noted that a threshold for low-frequency vibrational modes was set, with the peak frequency in the aqueous phase being 60 cm⁻¹, to avoid unreasonable large entropy contributions. Thus, low-frequency modes were reset to 60 cm⁻¹.

The CHE method was used to calculate the Gibbs free energy of the proton-electron pair (H^+^ + e^−^):

G(H^+^) + G(e^−^) = 1/2 G(H_2_) – eU

where G(H^+^) and G(e^−^) are the Gibbs free energies of the proton and electron, respectively; G(H_2_) is the Gibbs free energy of hydrogen gas under standard conditions; hence, U is the applied potential relative to the standard hydrogen electrode (SHE). Note that G(H_2_(g)) is calculated as an isolated gas molecule in a unit box, considering contributions from translational, rotational, and vibrational free energies under the ideal gas approximation. G(H_2_O(l))) is calculated similarly to G(H_2_(g)), but with adjustments for terms related to the saturation vapor pressure in equilibrium with liquid water at room temperature (p/p°=0.031). Finally, G(O_2_(g)) is derived from the following expression:

G(O_2_(g))+2G(H_2_(g))−2G(H_2_O(l))=4.92 eVG

where this value corresponds to the total reaction energy for the OER under standard conditions (SHE scale).

The OER mechanism proceeds through the following pathways, where the adsorption sites on bare metal cations are indicated (O_V_ represents lattice oxygen vacancies, and M can be iron). Furthermore, to facilitate differentiation between the reaction mechanisms, we annotated the composition of the final product O_2_ in the reaction pathways, including the source of oxygen. Each reaction equation corresponds to its respective reaction step in the free energy diagram, including ΔG.


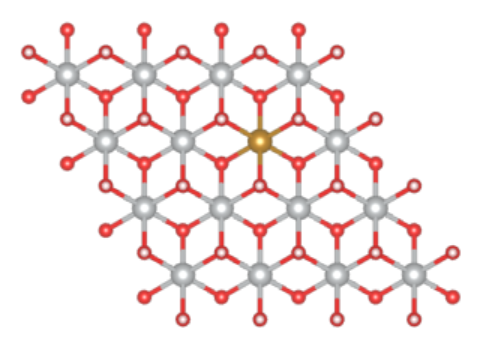


**Figure S1**. Fe-Ni(OH)_2_ model structure.


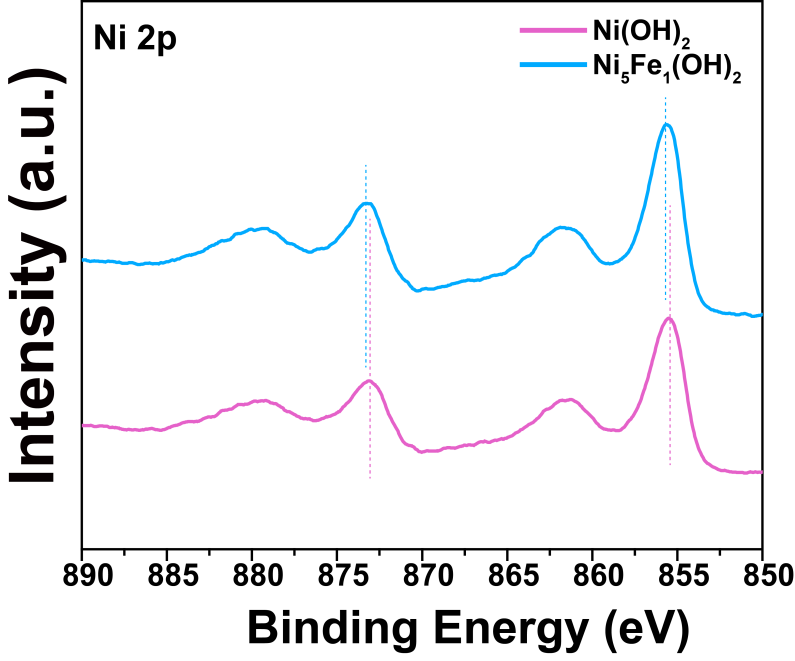


**Figure S2**. The high-resolution XPS of Ni 2p of the Ni(OH)_2_ and Ni_5_Fe_1_(OH)_2_.


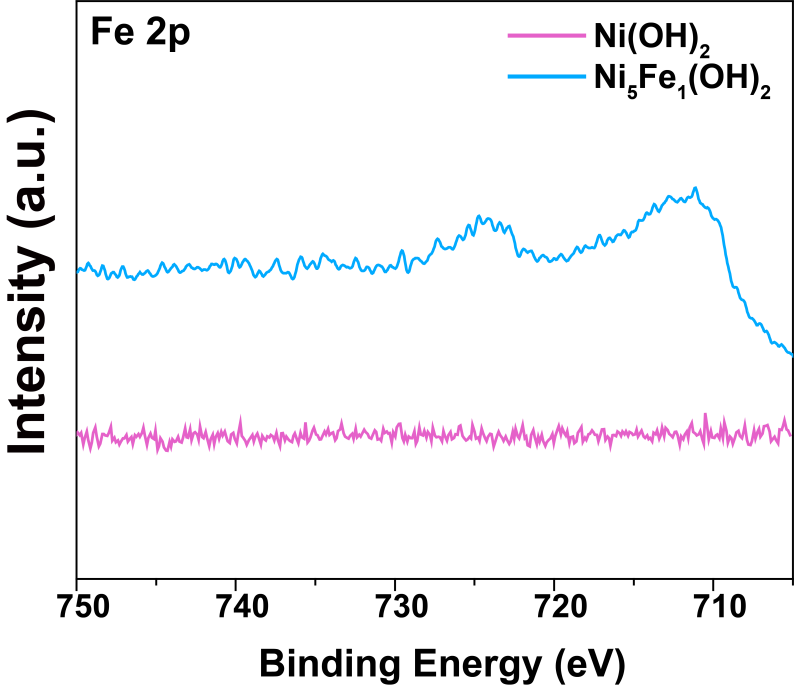


**Figure S3**. The high-resolution XPS of Fe 2p of the Ni(OH)_2_ and Ni_5_Fe_1_(OH)_2_.


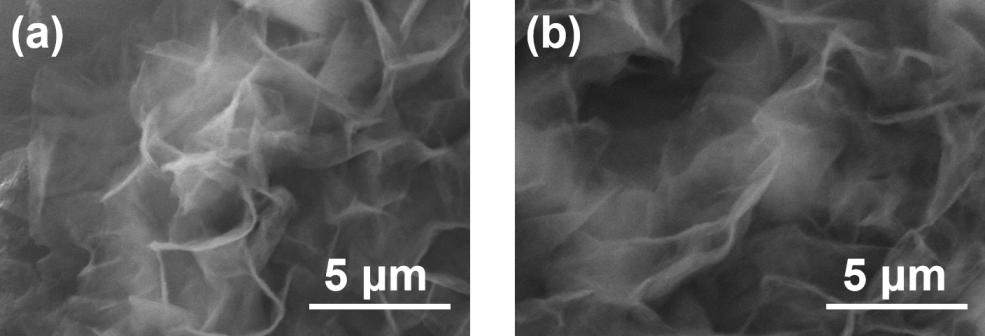


**Figure S4**. SEM image of (a) Ni(OH)_2_ and (b) Ni_5_Fe_1_(OH)_2_.


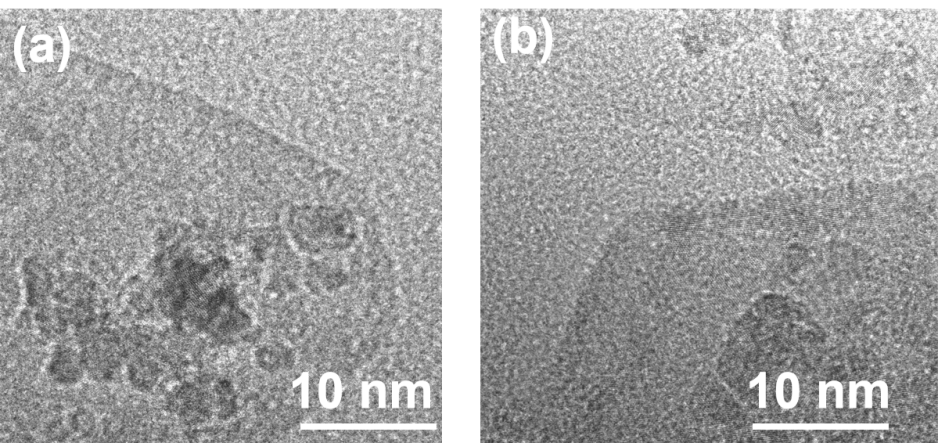


**Figure S5.** TEM image of (a) Ni(OH)_2_ and (b) Ni_5_Fe_1_(OH)_2_.


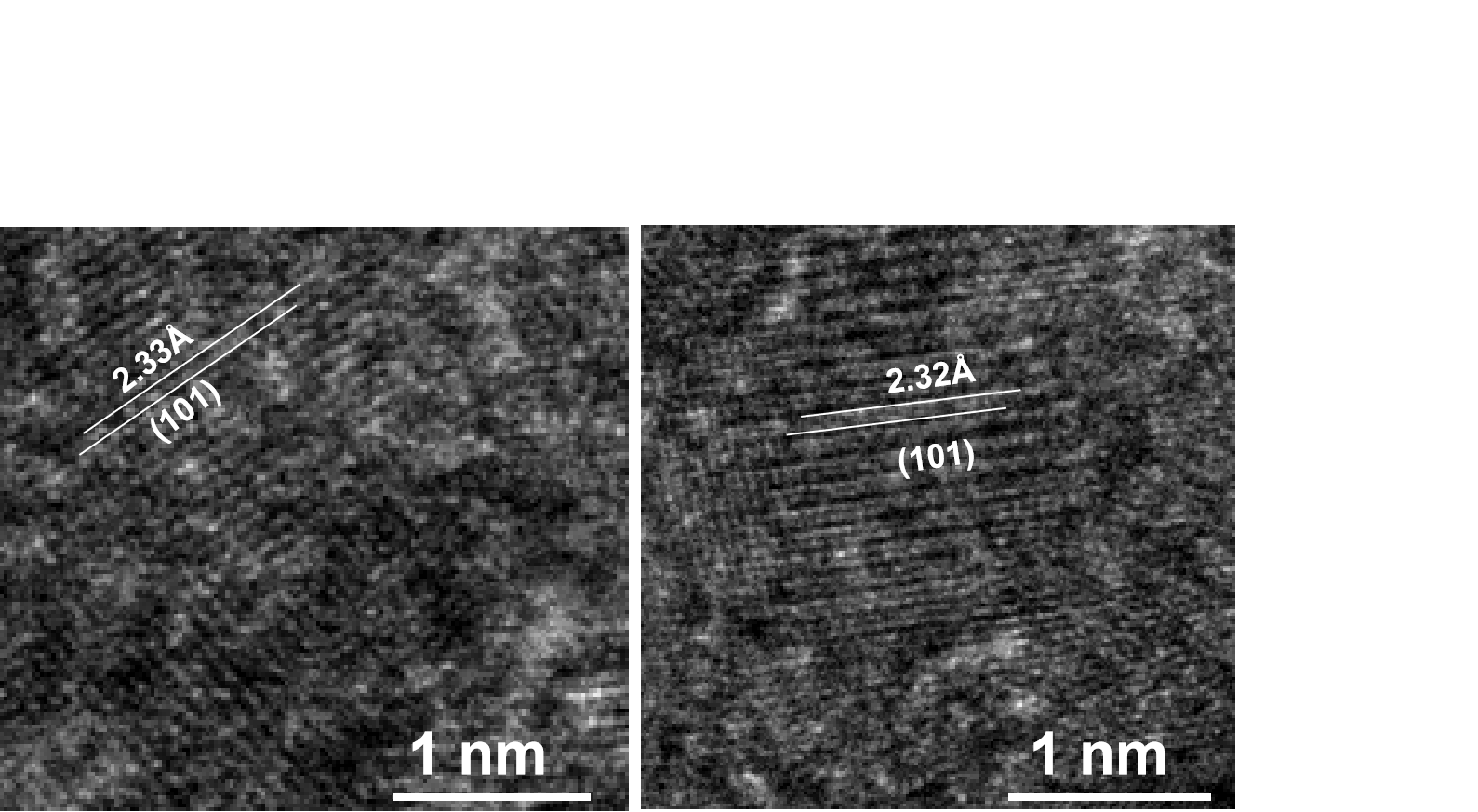


**Figure S6.** HRTEM image of Ni(OH)_2_.


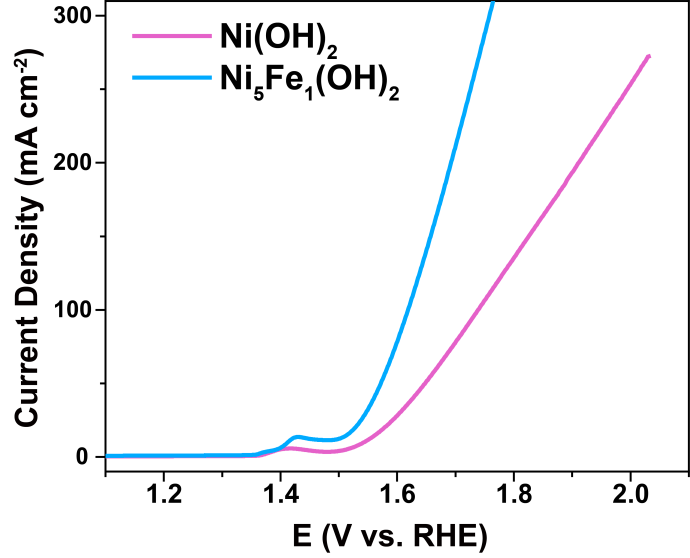


**Figure S7**. Polarization curves of Ni(OH)_2_ and Ni_5_Fe_1_(OH)_2_.


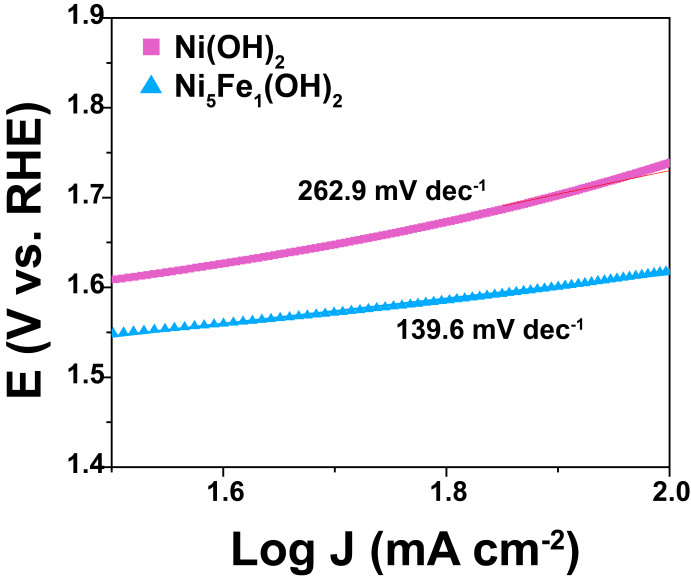


**Figure S8**. Tafel plots of Ni(OH)_2_ and Ni_5_Fe_1_(OH)_2_.

**
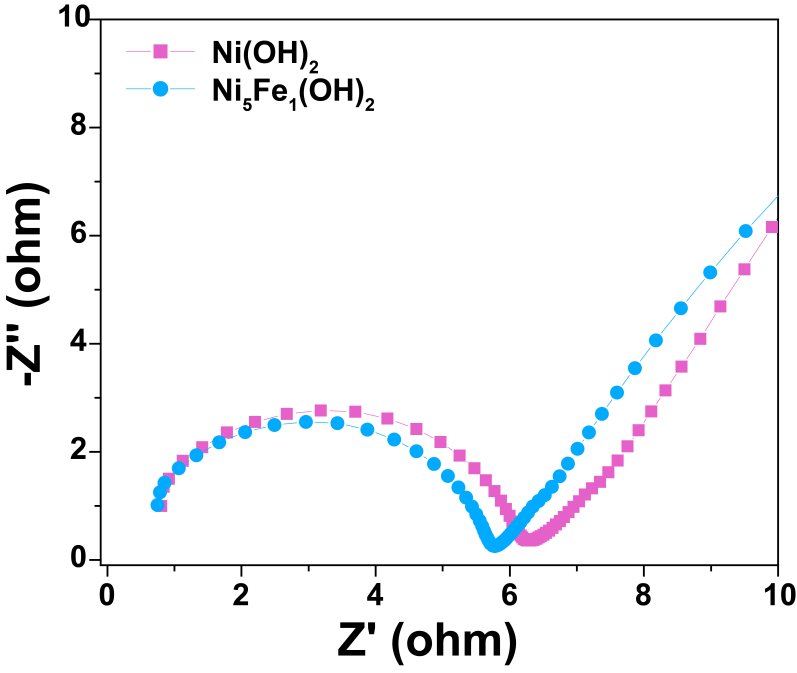
**

**Figure S9**. EIS of Ni(OH)_2_ and Ni_5_Fe_1_(OH)_2_.


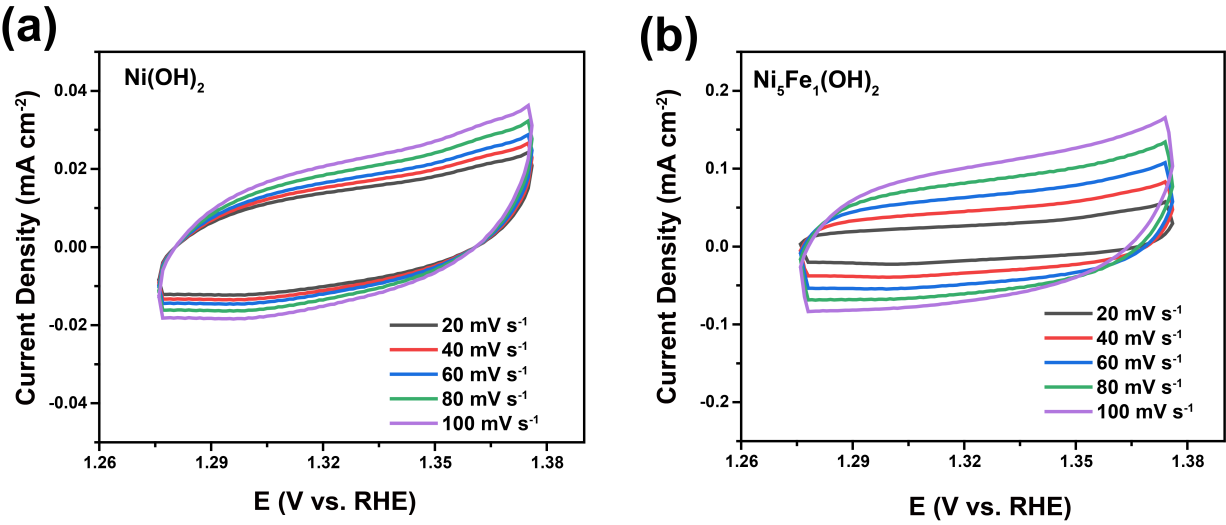


**Figure S10.** The CVs of Ni(OH)_2_ and Ni_5_Fe_1_(OH)_2_.

**
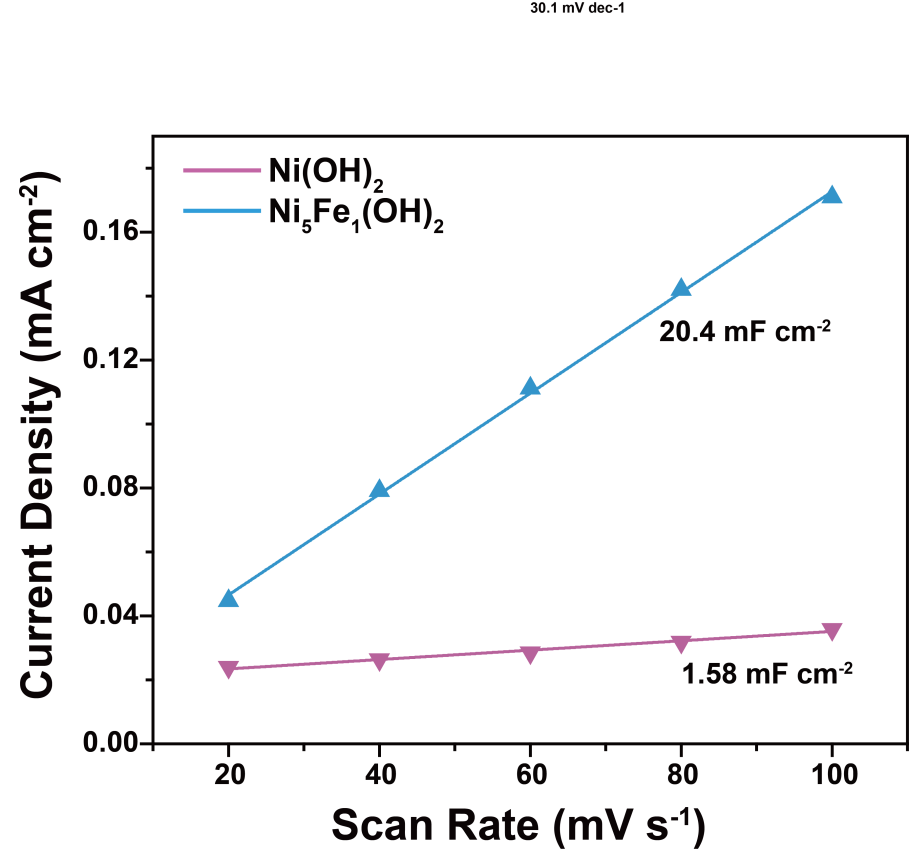
**

**Figure S11.** The ECSA of Ni(OH)_2_ and Ni_5_Fe_1_(OH)_2_.


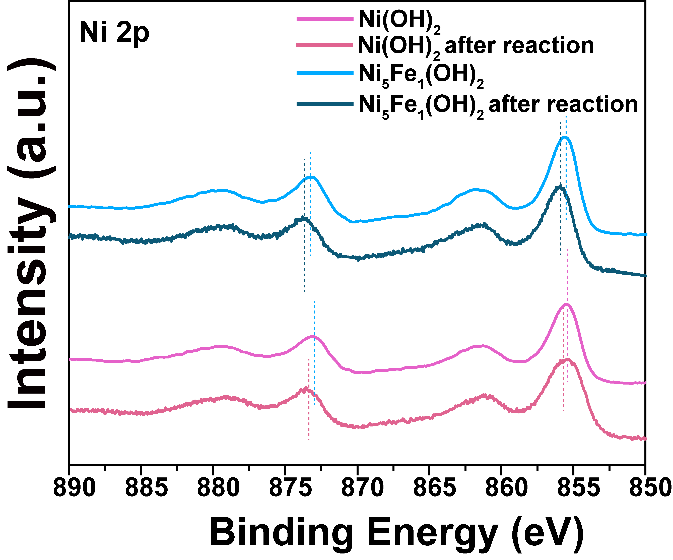


**Figure S12**. The high-resolution XPS of Ni 2p of the Ni(OH)_2_ and Ni_5_Fe_1_(OH)_2_ before and after LSV test.


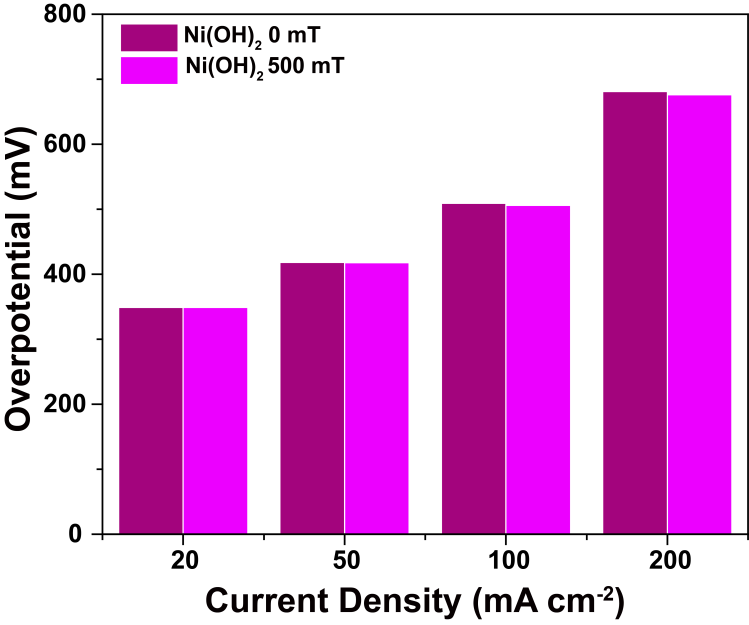


**Figure S13.** The overpotential of Ni(OH)_2_ under 0 mT and 500 mT at different potential.


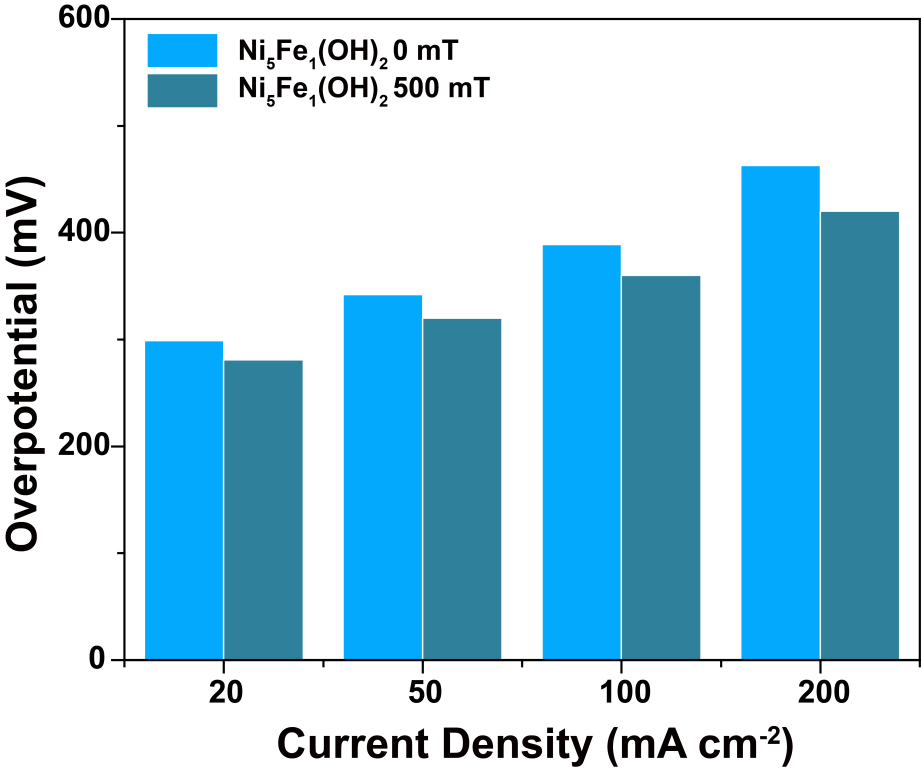


**Figure S14.** The overpotential of Ni_5_Fe_1_(OH)_2_ under 0 mT and 500 mT at different potentials.

**
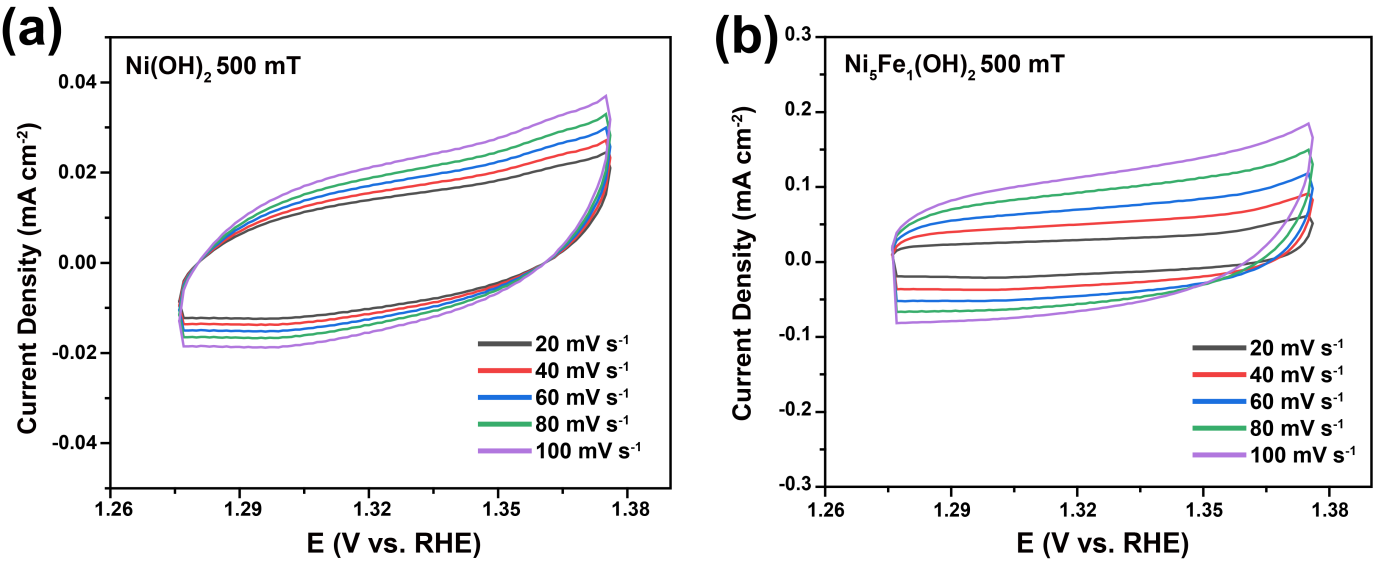
**

**Figure S15.** The CVs of Ni(OH)_2_ and Ni_5_Fe_1_(OH)_2_ under 500 mT.


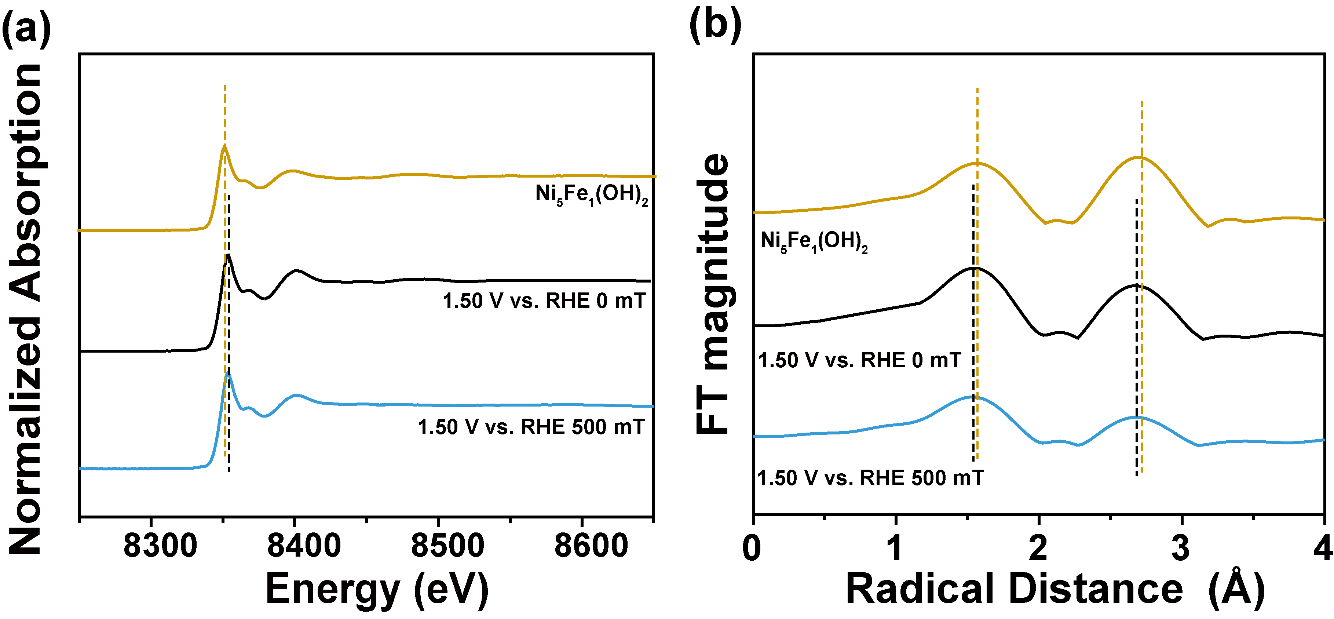


**Figure S16.** (a) XANES spectra and (d) Ni-K edge EXAFS spectra of the Ni-K edge on Ni_5_Fe_1_(OH)_2,_ Ni_5_Fe_1_(OH)_2_ at 1.5 V vs. RHE under 0 mT and Ni_5_Fe_1_(OH)_2_ at 1.5 V vs. RHE under 500 mT.

**
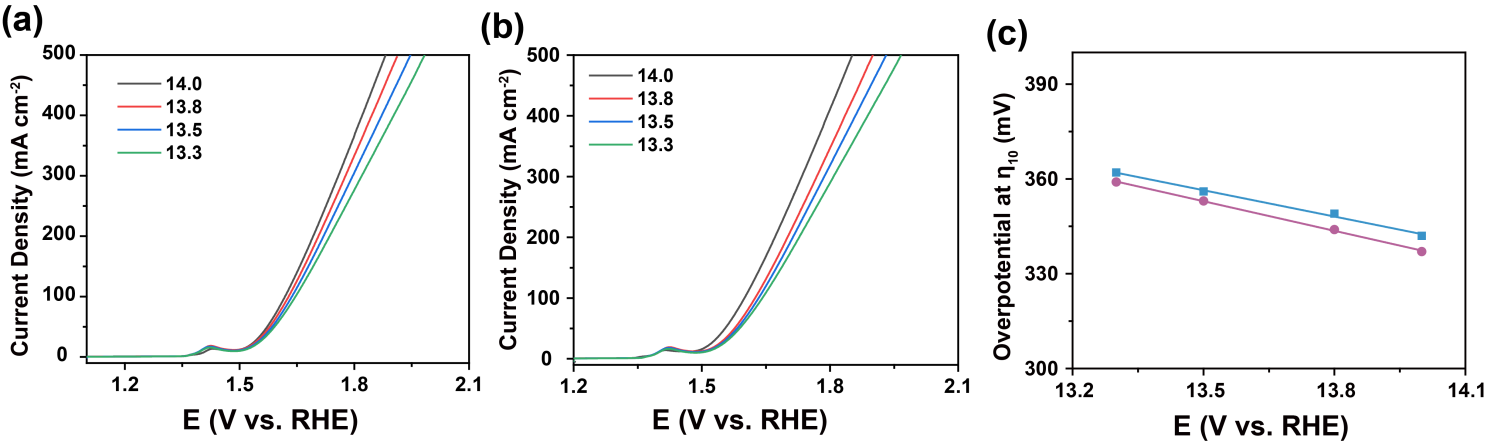
**

**Figure S17.** CV curves of Ni_5_Fe_1_(OH)_2_ under (a) 0 mT and (b) 500 mT in alkaline electrolytes with different pH. (c) OER current densities at 50 mA cm^-2^ overpotential with different pH value.

**Table S1.** ICP analysis data of Ni_5_Fe_1_(OH)_2_.

| Element | Atomic Percentage (At %) |
| --- | --- |
| Ni | 83.2 |
| Fe | 16.8 |
